# FLT3 inhibitors upregulate CXCR4 and E-selectin ligands via ERK suppression in AML cells and CXCR4/E-selectin inhibition enhances anti-leukemia efficacy of FLT3-targeted therapy in AML

**DOI:** 10.1038/s41375-023-01897-x

**Published:** 2023-04-21

**Authors:** Yannan Jia, Weiguo Zhang, Mahesh Basyal, Kyung Hee Chang, Lauren Ostermann, Jared K. Burks, Charlie Ly, Hong Mu-Mosley, Qi Zhang, Xin Han, William E. Fogler, John L. Magnani, Arnaud Lesegretain, Anna A. Zal, Tomasz Zal, Michael Andreeff

**Affiliations:** 1 Section of Molecular Hematology and Therapy, Department of Leukemia, The University of Texas MD Anderson Cancer Center, Houston, TX USA; 2 Blood Diseases Hospital & Institute of Hematology, Chinese Academy of Medical Sciences & Peking Union Medical College, Tianjin, China; 3 Department of Pathology/Laboratory Medicine, The University of Texas MD Anderson Cancer Center, Houston, TX USA; 4 GlycoMimetics, Inc., Rockville, MD USA; 5 Daiichi Sankyo, Inc., Basking Ridge, NJ USA; 6 Department of Leukemia, The University of Texas MD Anderson Cancer Center, Houston, TX USA

**Keywords:** Cancer therapeutic resistance, Translational research

## To the Editor:

Fms-like tyrosine kinase 3 inhibitor(s) (FLT3i), including sorafenib, quizartinib, and FDA-approved midostaurin and gilteritinib, are commonly used to treat adult acute myelogenous leukemia (AML) with *FLT3* internal tandem duplication (ITD) mutations. These mutations are present in about 30% of adult AML cases and are associated with a poor prognosis [1]. However, FLT3i are not very effective in eliminating leukemia blasts in bone marrow (BM) [2, 3], which suggests that interactions between AML blasts and the BM microenvironment (BMM) provide a sanctuary for AML cells and protect them from targeted therapies [4].

BMM or “niche”-mediated resistance may result from the influence of extrinsic cytokines or chemokines, such as CXC chemokine receptor 4 (CXCR4)/CXC motif ligand 12 (CXCL12) and endothelial (E)-selectin (CD62E)/E-selectin ligands (E-selectin-L). Both CXCR4 and E-selectin ligands play crucial roles in directing leukocyte or hematopoietic stem cell (HSC) homing to BMM [5, 6]. In addition, the hypoxic condition (with oxygen levels of 1–3%) in BMM can also trigger the upregulation of genes glycosylating E-selectin-L through the hypoxia-inducible factor 1-α—a key transcriptional factor induced by hypoxia, known to be involved in the synthesis of the carbohydrate ligands for E-selectin and is associated with AML therapy resistance [7, 8].

We hypothesized that concomitantly targeting CXCR4 and E-selectin could enhance the anti-leukemia efficacy and alleviate the BM niche-mediated resistance to FLT3-targeted therapy. If this hypothesis is validated, this concept could serve as a backbone for developing combinatorial strategies with FLT3i as a frontline therapy for FLT3-mutant AML.

**Fig. 1:**
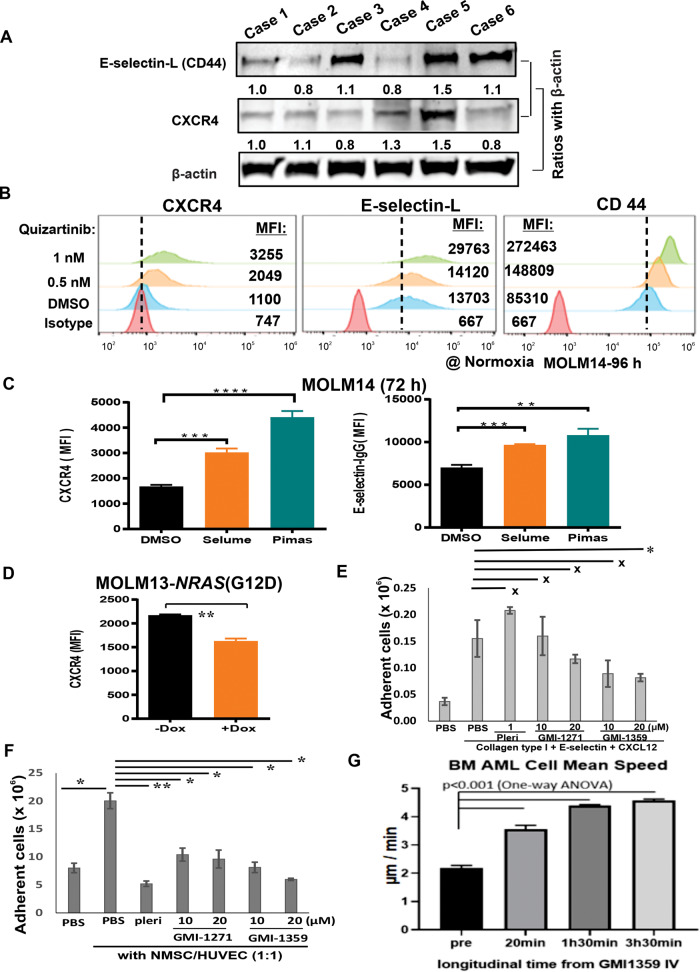
CXCR4 and E-selectin-L levels are associated with FLT3i resistance, which is upregulated by FLT3-targeted treatment through suppression of MAPK in *FLT3*-mutant AML cells. Blockade of CXCR4/E-selectin impairs leukemia cell adhesion/migration to BM components and increases leukemia cell mobility in BM. **A** Leukemia blasts collated from relapsed/refractory and newly diagnosed/complete remission AML patients were lysed with cell lysis buffer. CXCR4 and CD44 basal levels were determined with western blot. Semiquantitative immunoblotting data were generated using Odyssey software v2.0. β-actin was used as a loading control. **B** MOLM14 cells were exposed to FLT3i quizartinib for 96 h, and cell surface levels of CXCR4, E-selectin-L, and CD44 were determined by staining with anti-CXCR4-PE, E-selectin-IgG-PE, and anti-CD44-PE antibodies and median fluorescence intensity (MFI) was measured using flow cytometry. **C** MOLM14 cells were exposed to the MEK inhibitors selumetinib (400 nM) or pimasertib (80 nM) for 72 h, and MFI of cell surface CXCR4 and E-selectin-L were measured using flow cytometry. **D** CXCR4 level were determined by measuring MFI using flow cytometry after inducing NRAS (MAPK) overexpression in doxycycline (Dox)-inducible MOLM13-NRAS(G12D) cells by comparing with non-Dox induced cells. **E** Collagen type I, E-selectin, and CXCL12 were precoated onto 24-well plates overnight, washed with PBS, and blocked with 1% BSA. MOLM14 and the precoated wells were pretreated with the indicated drugs for 2 h. MOLM14-GFP cells (1 × 10^6^ cells/well) were seeded in triplicate into the wells for an additional 30-min culture. The wells were gently washed with PBS for 5 min on a shaker to remove unattached cells. The attached GFP^+^ cells were trypsinized and calculated using flow cytometry with counting beads. **F** Normal MSC (NMSC)/human umbilical vein endothelial cells (HUVEC) (1:1 ratio) were plated onto 24-well plates to reach 80% confluence. The NMSC/HUVEC and *FLT3*-ITD mutant MV4:11-GFP leukemia cells were pretreated with the indicated concentrations of plerixafor, GMI-1271, and GMI-1359 for 1 h. MV4:11 cells (0.5 × 10^6^ cells) were seeded into transwells (φ 0.1 µm) and co-cultured with NMSC cells/EC for an additional 16 h. The cells in the outer chambers were collected by trypsinization. GFP^+^ cells were counted using flow cytometry with counting beads. **G** GMI-1359 increases AML cell motility in the BM. C57BL6 mice (harboring CD11c-EYFP and hCD2-DsRed reporter genes, not shown) were implanted with AML1-mTurquoise cells. Mice with established AML (~5% blasts in circulation) were anesthetized and the calvarial BM was 3D imaged using time-lapse intravital 2-photon microscopy for 30 min before and for up to 3.5 h after GMI-1359 intravenous infusion. Cell motility in the BM was tracked and quantified using Imaris software at the indicated time points. All experiments were repeated at least three times, and data are presented as mean ± SD. The asterisks indicate the level of statistical significance. *p < 0.05; **p < 0.01; ***p < 0.001; ****p < 0.0001; and x = not statistically significant, as determined using two-tailed unpaired *t*-test or one-way ANOVA with Dunnett’s post hoc test. Selume selumetinib, Pimas pimasertib, Pleri plerixafor.

To this end, we first investigated whether the baseline levels of CXCR4/E-selectin-L were associated with FLT3i resistance. Results showed that leukemia blasts from relapsed/refractory *FLT3*-mutated AML patients exhibited higher levels of CXCR4 and CD44 (a major carrier of non-canonical E-selectin ligands) compared to those of newly diagnosed or complete remission patients (Fig. 1A and Supplementary Table 1). FLT3i resistant cells (i.e., Ba/F3 cells harboring-*FLT3*-ITD+D835Y and *FLT3*-ITD+F691L mutations) also demonstrated higher levels of CXCR4, E-selectin-L, and CD44 expression on their cell surface compared to FLT3i sensitive cells Ba/F3-*FLT3*-ITD cells (Supplementary Fig. S1a). These findings suggest that high levels of CXCR4/E-selectin-L may be linked to drug resistance in FLT3-targeted therapy.

Furthermore, we observed that the levels of CXCR4, E-selectin-L, and CD44 were upregulated in a dose- and time-dependent manner following 2–96 h of quizartinib (or sorafenib) treatment in *FLT3*-ITD mutant MOLM14 cells, and this upregulation was mediated at the transcriptional level (Fig. 1B and Supplementary Figs. S1b and S2–S4). As expected, we also found that hypoxia, one of the primary factors mediating resistance to AML therapy [9], upregulated the levels of CXCR4, and P-selectin glycoprotein ligand-1 in FLT3-ITD mutant leukemia cells (MOLM13, MOLM14, and MV4:11 cells) (Supplementary Fig. S5).

Interestingly, our study found that the upregulation of CXCR4 and E-selectin-L in MOLM14 cells was associated with the suppression of ERK, but not of AKT/mTOR or STAT5, as demonstrated by treatment with the specific MEK inhibitors selumetinib and pimasertib, instead of FLT3i (Fig. 1C and Supplementary Fig. S6). This suggests that the FLT3i-induced suppression of ERK is responsible for the observed upregulation of CXCR4 and E-selectin-L. On the contrary, the further upregulation of MAPK downregulates CXCR4 level in doxycycline-inducible MOLM13-*NRAS*^(G12D)^ cells (Fig. 1D and Supplementary Fig. S7).

Since CXCR4/CXCL12 and E-selectin/E-selectin-L axes are critical for leukemia cell adhesion/migration in the BM niche environment, we further assessed the effects of CXCR4 or E-selectin blockade on leukemia cell adhesion and migration to BM components (mesenchymal stem cells/endothelial cells or E-selectin/CXCL12 chemokines) using the specific small molecule CXCR4 inhibitors plerixafor and E-selectin inhibitor GMI-1271, and then comparing them with the dual CXCR4/E-selectin antagonist GMI-1359. GMI-1359 was found to be more effective than single inhibitors plerixafor or GMI-1271 in reducing leukemia cells adhesion and migration to BM components in normoxia and in hypoxia (Fig. 1E, F and Supplementary Fig. S8), and triggering much more apoptosis induction in leukemia cells while combination treatment of GMI-1359 with FLT3i quizartinib or FDA-approved midostaurin (Supplementary Fig. S9). These findings support the hypothesis that targeting CXCR4 and E-selectin could serve as a backbone for the development of combinatorial strategies with FLT3i as frontline therapy of *FLT3*-mutant AML.

Of note, leukemia cell mobility in the BM increased profoundly in mice BM compared with control vehicle-treated mice after one dose of GMI-1359 in a time-dependent manner (from 20 min to 4 h after drug infusion), as determined by intravital 2-photon laser scanning microscopy of intravasation and cellular outflow through the BM capillary vasculature. GMI-1359 infusion resulted in a 66% increase in the average speed of cellular motility of AML cells within 20 min and a >100% increase within 3.5 h (5.4 µm/min) (Fig. 1G). The findings suggest that blockade of E-selectin/CXCR4 with GMI-1359 enhances the dynamic activity of leukemia cells and enables them to detach from the BM niche environment by disrupting the binding of leukemia cells to niche cells, resulting in an increase in circulating leukemia cells in vivo (Fig. 1G and Supplementary Video).

**Fig. 2:**
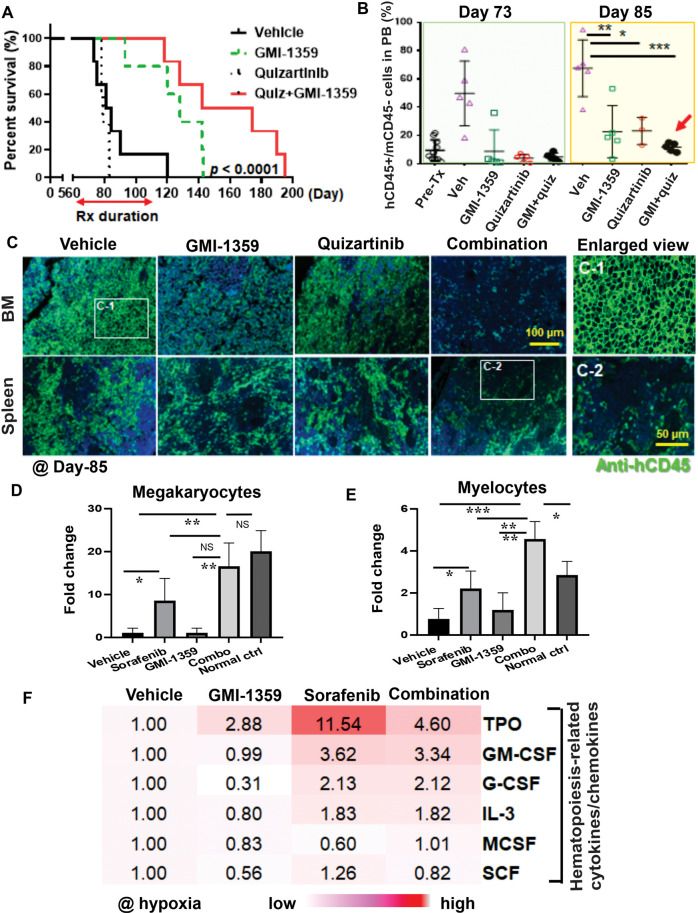
Co-targeting CXCR4/E-selectin and FLT3 with GMI-1359 and quizartinib reduces leukemia burden and extends survival in PDX leukemia models. As well as the combination of GMI-1359 and sorafenib improves mouse normal hematopoiesis with upregulation of hematopoiesis-related cytokines. **A** NSG mice xenografted with *FLT3*-ITD mutant PDX AML cells received quizartinib and/or GMI-1359, and mouse survival duration was estimated using the Kaplan–Meier method. The arrow bar indicates the treatment duration. Rx = treatment. **B** Leukemia cell engraftment in peripheral blood was measured by flow cytometry on days 73 and 85, respectively. **C** Leukemia cell burdens in the BM and spleen were observed by sacrificing three mice in each group at day 85 and staining the histological sections with anti-hCD45 antibodies. Far-right two figures show an enlarged view and indicate hCD45-positive cells from the BM (C-1) and negative cells from the spleen (C-2). **D**, **E** NSG mice engrafted with PDX AML cells received sorafenib and/or GMI-1359 (53 days of treatment), and BM histological sections were stained with anti-mouse CD41 and anti-mouse CD13 antibodies. Semiquantitative analysis was performed by counting megakaryocytes (mCD41+) and myelocytes (mCD13+) cells. The numbers on *y*-axes indicated the fold changes to cell numbers in the vehicle group which was counted as 1. Error bars are presented as the means ± standard deviation. **F** MOLM14 cells were cultured with MSC/EC for 24 h in hypoxia condition after being pretreated with GMI-1359 for 1 h. Hematopoiesis-related cytokines were analyzed using the Human Cytokine Antibody Array (ab133997, Abcam, Boston, MA) following the manufacturer’s instructions. The numbers were shown in folds by comparing the levels in vehicle media, which was counted as 1. The color scale from 0 (white) to 12 (red) (units in mean normalized spot density). The asterisks indicate the level of statistical significance. *p < 0.05; **p < 0.01; ***p < 0.001; NS not statistically significant.

Moreover, co-targeting CXCR4/E-selectin and FLT3 with the combination of GMI-1359 and quizartinib demonstrated significant anti-leukemia efficacy in a patient-derived xenograft (PDX) model (xenografted with *FLT3*-mutant AML blasts from a relapsed patient who had received sorafenib-targeted therapy), resulting in prolonged survival of the mice compared to vehicle controls or single-agent treatments (median survival, 158 vs. 82.5, 79, and 128 days, respectively, in the combination group vs. vehicle, quizartinib, or GMI-1359; *p* < 0.0001) (Fig. 2A), and was accompanied with profound reduction of leukemia burden in circulation, BM and spleen (Fig. 2B, C). These findings suggest that the co-targeting of CXCR4/E-selectin and FLT3 with the combination of GMI-1359 and quizartinib may represent a promising therapeutic strategy for the treatment of *FLT3*-mutated AML.

Interestingly, we found that a combination of GMI-1359 and FLT3i sorafenib improved the recovery (or protection) of mouse normal hematopoiesis in the AML PDX model, with a significant increase in megakaryocytes and myelocytes in the BM, as evidenced by histological analysis (H&E staining) (Supplementary Fig. S10). Furthermore, we labeled megakaryocytes and myelocytes with anti-mouse CD41 and CD13 antibodies, respectively, and observed an increase in megakaryocytes (8.5-fold, *p* < 0.05) and myelocytes (2.23-fold, *p* < 0.05) in the sorafenib-alone treatment group compared with in the vehicle group. However, a much higher increase in megakaryocytes (16.5-fold, *p* < 0.01) and myelocytes (4.52-fold, *p* < 0.001) was observed in the combination therapy compared to the vehicle group in the BM (Fig. 2D, E and Supplementary Fig. S11a, b). These results strongly suggest that co-targeting CXCR4/E-selectin/FLT3 (with sorafenib) may be a promising approach for AML treatment with potential protective effects on normal hematopoiesis of mouse BM.

We have previously reported in phase 2a clinical study that targeting CXCR4 with the antagonist BL-8040 achieved significant mobilization of blasts into circulation, accompanied by a decrease in BM blasts [10]. These findings also suggest that GMI-1359, in combination with FLT3i, may be a potential therapeutic strategy for patients with *FLT3*-mutant AML. This could potentially overcome secondary resistance to FLT3-targeted therapy, which may involve the upregulation of surface CXCR4/E-selectin-L in AML cells. Further clinical studies are needed to evaluate the safety and efficacy of this combination therapy in AML patients.

Myelosuppression represents a major dose-limiting toxicity in chemo or targeted therapies [11]. Normal hematopoiesis can be augmented by many cytokines, such as TPO, GM-CSF, G-CSF, and IL-3, which enhance HSCs differentiation and improve the efficacy of chemotherapy and targeted therapy in leukemia patients [12–14]. We observed that the combination of GMI-1359 and sorafenib resulted in an increase of TPO, GM-CSF, G-CSF, and IL-3 levels after exposure to sorafenib or the combination of sorafenib/GMI-1359 in hypoxia for 24 h (Fig. 2F and Supplementary Fig. S12), suggesting that may be beneficial for the recovery of normal hematopoiesis which may result from the cytokine-mediated differentiation of HSCs, evidenced as an increase of murine myelocytes and megakaryocytes in the BM of the PDX model after the treatment (Fig. 2D, E). However, it should be noted that this effect was not observed with quizartinib (data not shown), which may have off-target toxicities that impair normal hematopoiesis by its c-Kit inhibition [15]. Further research is needed to explore the potential benefits and limitations of combining CXCR4/E-selectin inhibitors with different FLT3i in the treatment of *FLT3*-mutant AML.

Indeed, further investigation into the effects of co-targeting CXCR4/E-selectin/FLT3 on normal hematopoiesis and chemokine/cytokine profiles in the BMM scenario would be crucial for better understanding the protective mechanisms of normal hematopoiesis by CXCR4/E-selectin inhibition during FLT3-targeted therapy. Future clinical trials could help shed more light on these mechanisms and provide insights into potential strategies to mitigate myelosuppression while improving the efficacy of targeted therapies for AML. Overall, this study provides important pre-clinical evidence for the potential of co-targeting CXCR4/E-selectin/FLT3 in *FLT3*-mutant AML, and highlights the importance of further investigating the underlying mechanisms of action in both leukemia cells and normal hematopoietic cells.

In summary, CXCR4/CXCL12 and E-selectin/E-selectin-L axes play critical roles in leukemia cell homing to the BMM niche and are closely associated with resistance to FLT3-targeted therapy in *FLT3*-mutant AML patients. We here report for the first time that CXCR4/E-selectin-L are transcriptionally upregulated by FLT3 inhibition, which was associated with suppression of MAPK signaling, and nuclear translocation of phospho-ERK may be involved in CXCR4 transcriptional regulation (Supplementary Fig. S13). Concomitant blockade of CXCR4/E-selectin with the dual inhibitor GMI-1359 disrupts leukemia cell homing/migration to BM niches. Combination treatment with GMI-1359 and quizartinib significantly reduces the leukemia burden and extends mouse survival in a PDX AML model, and the combination of GMI-1359 with sorafenib benefits the recovery of normal hematopoiesis. These findings provide pre-clinical rationale for combined CXCR4/E-selectin/FLT3 targeting in *FLT3*-mutant AML.

## Supplementary information

Supplementary video for observing cell mobility

Supplementary materials and methods

Supplementary figures

## Data Availability

The data that support the findings of this study are available from the corresponding author upon reasonable request.
