# Supplementary material for: FLT3 inhibitors upregulate CXCR4 and E-selectin ligands via ERK suppression in AML cells and CXCR4/E-selectin inhibition enhances anti-leukemia efficacy of FLT3-targeted therapy in AML
Source: Leukemia. 2023 Apr 21;37(6):1379–83. doi: 10.1038/s41375-023-01897-x (PMC10244167; doi:10.1038/s41375-023-01897-x)
Supplement: Supplementary file 1 — Supplementary video for observing cell mobility [file 41375_2023_1897_MOESM1_ESM.pptx]

## Slide 1
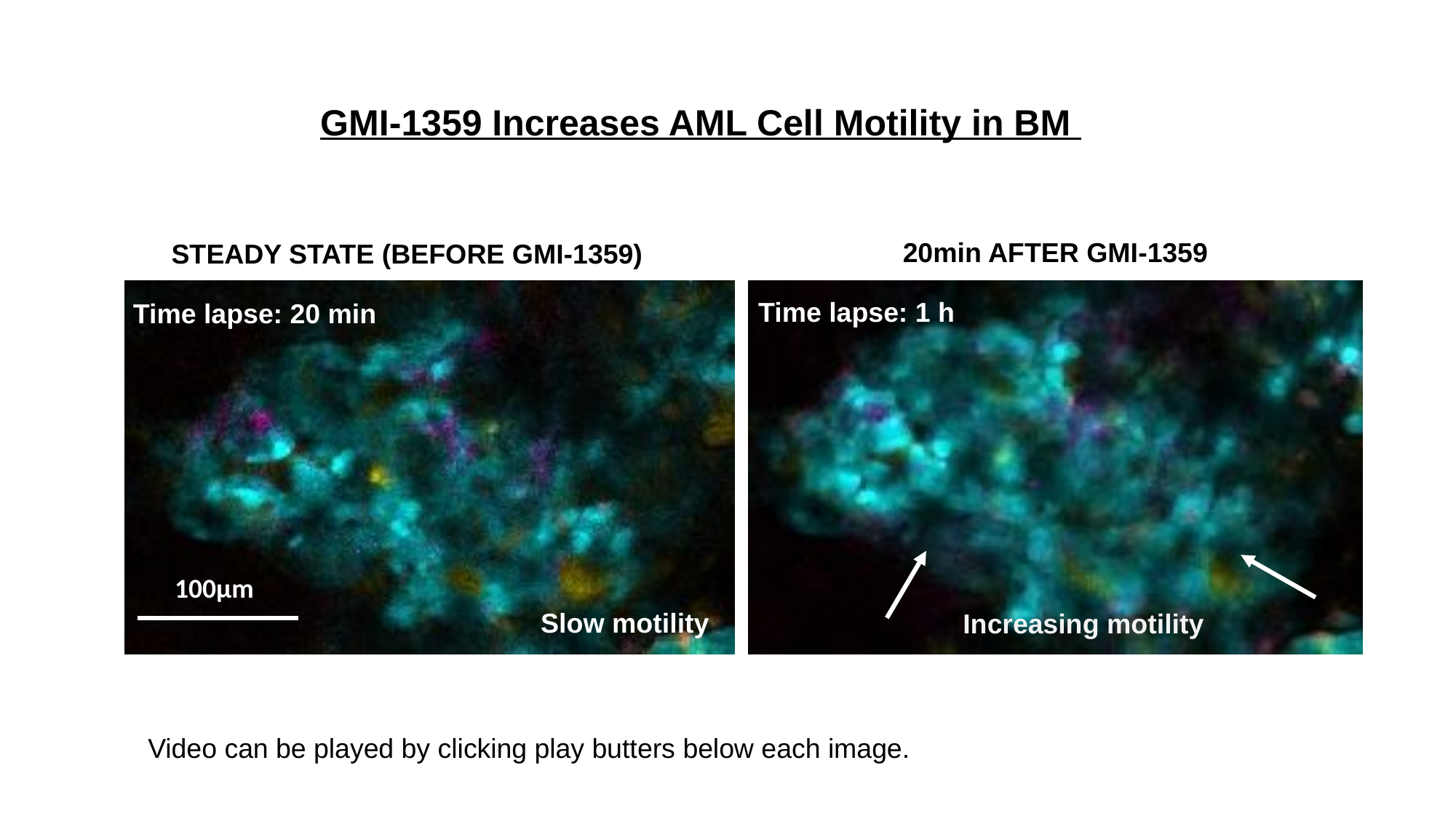

GMI-1359 Increases AML Cell Motility in BM
20min AFTER GMI-1359
STEADY STATE (BEFORE GMI-1359)
Time lapse: 1 h
Time lapse: 20 min
100µm
Slow motility
Increasing motility
Video can be played by clicking play butters below each image.
