# Supplementary material for: FLT3 inhibitors upregulate CXCR4 and E-selectin ligands via ERK suppression in AML cells and CXCR4/E-selectin inhibition enhances anti-leukemia efficacy of FLT3-targeted therapy in AML
Source: Leukemia. 2023 Apr 21;37(6):1379–83. doi: 10.1038/s41375-023-01897-x (PMC10244167; doi:10.1038/s41375-023-01897-x)
Supplement: Supplementary file 2 — Supplementary materials and methods [file 41375_2023_1897_MOESM2_ESM.docx]

**Supplementary Methods:**

### Compounds and chemokines

The FLT3i sorafenib, quizartinib, midostaurin, and the MEK inhibitors selumetinib and pimasertinib were purchased from Selleckchem (Houston, TX). The CXCR4 inhibitor plerixafor was from The University of Texas MD Anderson Cancer Center Pharmacy Store (Houston, TX). The E-selectin inhibitor GMI-1271 and CXCR4/E-selectin inhibitor GMI-1359 were kindly provided by GlycoMimetics, Inc. ([Rockville, MD](https://www.google.com/search?sxsrf=AOaemvIMurRE7GMIVNvlyCx3R64-t0Qr-Q:1638829157985&q=Rockville,+Maryland&stick=H4sIAAAAAAAAAOPgE-LSz9U3MCq2tIzPUuIAsZMqkpK0tLKTrfTzi9IT8zKrEksy8_NQOFYZqYkphaWJRSWpRcWLWIWD8pOzyzJzclJ1FHwTiypzEvNSdrAyAgDwlYwYXQAAAA&sa=X&ved=2ahUKEwi_kbyumtD0AhXzkGoFHZ_bBXoQmxMoAXoECDwQAw), USA). CXCL12 and E-selectin were from R&D Systems ([Minneapolis, MN](https://www.google.com/search?sxsrf=AOaemvL4jPymZb5hPxWsa4gd-Qx0XcE8cg:1638830131615&q=Minneapolis&stick=H4sIAAAAAAAAAOPgE-LVT9c3NExKNk02rMiqUOLUz9U3SCuoKk_T0spOttLPL0pPzMusSizJzM9D4VhlpCamFJYmFpWkFhUvYuX2zczLS00syM_JLN7ByggA7JP4C1kAAAA&sa=X&ved=2ahUKEwik2t3-ndD0AhWhkWoFHVYsBC4QmxMoAXoECEUQAw)). Collagen type I was from BD Bioscience (San Jose, CA).

**Cell lines and culture**

Ba/F3-*FLT3*-ITD+D835Y and Ba/F3-*FLT3*-ITD+F691L cells, which bear *FLT3* ITD and TKD point mutations, were generated as described previously ^1^.

The human MOLM13 and MOLM14 AML cell lines were purchased from the Deutsche Sammlung von Mikroorganismen und Zellkulturen (Braunschweig, Germany), and the MV4:11 cell line was from ATCC ([Manassas, VA](https://www.google.com/search?sxsrf=APq-WBtJ8viD8Y5l7spATZwadbFz0Z6unA:1646155609671&q=Manassas&stick=H4sIAAAAAAAAAOPgE-LUz9U3MC-MNytU4gAxc_OyTLSMMsqt9JPzc3JSk0sy8_P084vSE_MyqxJBnGKrjNTElMLSxKKS1KJihZz8ZLDwIlYO38S8xOLixOIdrIwAt0xpB1kAAAA&sa=X&ved=2ahUKEwikqu_Ct6X2AhUVm2oFHcoZCPwQmxMoAHoECEIQAg)). The Ba/F3-*FLT3*-ITD murine leukemia cell line was kindly provided by Dr. J. Donald Small (Department of Pediatric Oncology, Johns Hopkins University, Baltimore, MD). All details of the engineered cell lines were derived from the above cell lines and BM niche cell lines (i.e. MSC and EC); the culture conditions are provided in the Supplementary Methods. MOLM13-*NRAS* cells contain the *NRAS^G12D^* mutant and are doxycycline-inducible, as described previously ^2^. *NRAS^G12D^* expression was induced with doxycycline (1 µg/ml) for 72 hours prior to the experiment. MOLM14-firefly-copGFP, MOLM14-GFP, and MV4:11-GFP cell lines were established by lentivirally introducing firefly/Cop-GFP or GFP into the cells, and GFP-positive cell populations were collected using fluorescence-activated cell sorting (FACSAria II, BD Bioscience, San Jose, CA).

Human normal MSCs (NMSCs) were obtained from healthy BM donors as described previously ^3^. The human BM stroma cell line HS-27A and the endothelial cell lines HUVEC and HEBC-5i were purchased from ATCC ([Manassas, VA](https://www.google.com/search?sxsrf=APq-WBtJ8viD8Y5l7spATZwadbFz0Z6unA:1646155609671&q=Manassas&stick=H4sIAAAAAAAAAOPgE-LUz9U3MC-MNytU4gAxc_OyTLSMMsqt9JPzc3JSk0sy8_P084vSE_MyqxJBnGKrjNTElMLSxKKS1KJihZz8ZLDwIlYO38S8xOLixOIdrIwAt0xpB1kAAAA&sa=X&ved=2ahUKEwikqu_Ct6X2AhUVm2oFHcoZCPwQmxMoAHoECEIQAg)). Cell lines were maintained in RPMI medium supplemented with 10% fetal bovine serum, or according to the cell lines supplier’s instructions.

All cell lines were tested and authenticated by STR DNA fingerprinting, performed by the Characterized Cell Line Core Facility at MD Anderson (Houston, TX).

### Antibodies

### The antibodies used for FACS were as described below: mouse anti-human CD45, rat anti-mouse CD45, and mouse anti-human CD34 were from BD Biosciences (Franklin Lakes, NJ). Anti-human CD44-PE antibody was from Biolegend (San Diego, CA), anti-human CD184-PE was from BD Bioscience (San Jose, CA), and recombinant E-selectin-IgG-PE (which is a recombinant E-selectin-IgG-PE-conjugated fusion protein that recognizes E-selectin ligands on the cell surface, including CD44) was kindly provided by GlycoMimetics ([Rockville, MD](https://www.google.com/search?sxsrf=AOaemvIMurRE7GMIVNvlyCx3R64-t0Qr-Q:1638829157985&q=Rockville,+Maryland&stick=H4sIAAAAAAAAAOPgE-LSz9U3MCq2tIzPUuIAsZMqkpK0tLKTrfTzi9IT8zKrEksy8_NQOFYZqYkphaWJRSWpRcWLWIWD8pOzyzJzclJ1FHwTiypzEvNSdrAyAgDwlYwYXQAAAA&sa=X&ved=2ahUKEwi_kbyumtD0AhXzkGoFHZ_bBXoQmxMoAXoECDwQAw)).

All antibodies for immunoblotting are commercially available. Rabbit polyclonal antibodies were purchased from the following sources: phospho-FLT3 (Tyr589/591), phospho-ERK (Thr202/Tyr204), phospho-AKT [(Ser473)](http://www.cellsignal.com/products/4060.html), phospho-S6K (Ser240/Ser244), phospho-PI3K, and S6K were from Cell Signaling Technology (Beverly, MA); FLT3 and anti-N-Ras antibody (F155) were from Santa Cruz Biotechnology (Santa Cruz, CA); Stat5A/B was from R&D Systems (Minneapolis, MN); and CXCR4 was from Abcam (Boston, MA). Mouse monoclonal antibodies were purchased from the following sources: phospho-Stat5 (Tyr694/699) was from Upstate, Inc. (Charlottesville, VA), and ERK2 was from Santa Cruz Biotechnology (Santa Cruz, CA); tubulin and GAPDH were from Sigma-Aldrich (St. Louis, MO). **Apoptosis assays**

Cells were pretreated with plerixafor, GMI-1271 or GMI-1359 for 1-2 h and treated with FLT3i for an additional 48 h. Cells were harvested and apoptosis induction was determined by counting the Annexin V+/CD90- DAPI+ population via fluorescence-activated cell sorting and analyzed using the Gallios Flow Cytometer (Beckman Coulter), as described previously ^4^.

**Immunoblotting**

Immunoblotting was conducted as previously described ^5^. Phosphorylation and total protein levels were determined using the Odyssey Infrared Imaging System (LI-COR Biosciences, Lincoln, NE); semi-quantitative immunoblotting data were generated using Odyssey software v2.0. Tubulin or GAPDH was used as a loading control.

**Immunofluorescence staining**

Mouse tissues were harvested at the indicated time points and fixed with 10% neutralized formalin (pH 7.0). Bone tissue (femurs) was further decalcified with 14% EDTA buffer for an additional 5 days. All of the tissues were paraffin-embedded and sectioned. The sections (5-µm thick) were stained with H&E and analyzed by light microscopy. For immunofluorescence staining, the tissue sections were obtained via heat-induced epitope retrieval in sodium citrate buffer (10 mM sodium citrate, 0.05% Tween 20 [pH 6.0]) and incubated with anti-human CD45-Alex 488-conjugated antibody (M0701, Dako) overnight at 4°C. For the hematopoiesis analysis, the histological sections from the sorafenib or GMI-1359 combination-treated mice were stained with anti-mouse CD41 antibody (for labeling megakaryocytes) and anti-mouse CD13 antibody (for labeling myelocytes). Secondary anti-mouse IgG antibodies were conjugated with DyLight 649 or DyLight 488 (Vector), respectively, for 1 h. Counterstaining was performed with DABI and mounted with mounting media. The images were analyzed using fluorescence microscopy. A semi-quantitative analysis of immunofluorescence staining of mCD41 and mCD13 was performed by counting mCD41- and mCD13-positive cells under a 20x objective microscope lens (six random view fields per slide were chosen for counting).

**Quantitative polymerase chain reaction**

Total RNA was extracted using the RNeasy Plus Mini Kit (Qiagen). Reverse transcription was carried out using a High-Capacity cDNA Reverse Transcription Kit (Invitrogen). Quantitative polymerase chain reaction (qPCR) was performed using a QuantStudio 3 Real-Time PCR System with SYBR Green. The PCR reactions were set up on a 96-well optical plate by adding the following reagents into each well: 4 μl of cDNA and 10 μl of SYBR Green PCR Master Mix (Applied Biosystems, Foster City, CA, USA); the final concentrations of primers were 0.5 μmol/L in a final volume of 20 μl. The PCR amplification protocol was initiated at 50°C for 2 min, followed by 3 min at 95°C, 40 PCR cycles of 5 seconds at 95°C, and 60°C for 30 seconds. All samples were tested with the reference gene GAPDH for data normalization to correct for variations in RNA quality and quantity. The following primers were used for measuring gene transcriptional levels: GAPDH (forward 5’-GGAGCGAGATCCCTCCAAAAT-3’ and reverse 5’-GGCTGTTGTCATACTTCTCATGG-3’), CXCR4 (forward 5’-ACTACACCGAGGAAATGGGCT-3’ and reverse 5’-CCCACAATGCCAGTTAAGAAGA-3’), CD44 (forward 5’-CTGCCGCTTTGCAGGTGTA-3’ and reverse 5’-CATTGTGGGCAAGGTGCTATT-3’), and SELE (forward 5’-AGAGTGGAGCCTGGTCTTACA-3’ and reverse 5’-CTTTGCTGACAATAAGCACTGG-3’). The abundance of each transcript relative to GAPDH was calculated using the 2−ΔCt method, where ΔCt is the mean Ct of the transcript of interest minus the mean Ct of the transcript for GAPDH housekeeping gene.

**Adhesion assay**

MSC and EC were seeded in 12-well plates for growing to 70% confluence and then treated with TNFα (100 ng/mL) for 24 h. The cells were pretreated with plerixafor, GMI-1271, or GMI-1359 for 2 h and MOLM14-GFP cells (2x10^6^ cells/well) were added for an additional 20 h of co-culture in normoxia or hypoxia conditions. After being gently washed with PBS twice and then trypsinized, the adhered GFP+ cells were calculated using flow cytometry with counting beads.

Collagen type I, E-selectin, and CXCL12 were precoated onto 24-well plates overnight, washed with PBS, and blocked with 1% BSA. MOLM14 and the precoated wells were pretreated with the indicated drugs for 2 h. MOLM14-GFP cells (1x10^6^ cells/well) were seeded in triplicate into the wells for an additional 30-min culture. The wells were gently washed with PBS for 5 min on a shaker to remove unattached cells. The attached GFP+ cells were trypsinized and calculated using flow cytometry with counting beads.

**Migration assay**

Precoated normal MSC (NMSC)/human umbilical vein endothelial cells (HUVEC) (1:1 ratio) were plated onto 24-well plates to reach 80% confluence. The NMSC/EC (HUVEC) and MV4:11-GFP leukemia cells were pretreated with the indicated concentrations of plerixafor, GMI-1271, and GMI-1359 for 1 h. MV4:11 cells (0.5x10^6^ cells) were seeded into transwells (φ 0.1 µm) and co-cultured them with NMSC cells/EC for an additional 16 h. The cells in the outer chambers were collected by trypsinization. GFP+ cells were counted using flow cytometry with counting beads.

Instead of NMSC cells/EC, the BM niche components E-selectin (1 µg/mL), CXCL12 (0.1 µg/mL), and 10% FCS were added into the outer chambers. MV4:11-GFP leukemia cells were pretreated with plerixafor (2 µM), GMI-1271 (20 µM), and GMI-1359 (20 µM) for 1 h. The MV4:11 cells (1x10^6^ cells) were seeded into inner chambers for another 3 h. The cells in the outer chambers were calculated using flow cytometry after adding counting beads.

**Intravital 2-photon microscopy**

We used intravital 2-photon microscopy of calvarial BM to study the behavioral response of AML cells to the dual CXCR4 and E-selectin inhibition with GMI-1359 *in vivo*. We used, in triplicate, an mTurquoise2 fluorescence-tagged C57BL6 transplantable mouse AML model (AML1-mTurquoise; characterized by MLL, ENL-*FLT3*, ITD, p53^-/-^). To delineate the bone and vascular niches, the bone collagen and blood were highlighted by, respectively, second harmonic generation (SHG) and fluorescent TRITC-dextran. Intravenously infused AML1-mTurquoise cells (5x10^4^) homed to BM where they gradually displaced most endogenous cells. In this experimental system, GMI-1359 or vehicle were infused into the tail vein while recording AML cell motilities in calvarial BM stroma in 3-D over 4 h.

**Cytokine array**

HS-27A/HEBC-5i (at a 1:1 ratio) were seeded in 100-mm culture dishes for growing to 70% confluence. The MSC and EC and pretreated MOLM14 cells were treated with GMI-1359 for 1 h; MOLM14 cells were seeded on the MSC and EC feeder layer (at a 5:2 ratio) for an additional 24-h co-culture in the presence of sorafenib or GMI-1359 in hypoxia condition. DMSO was used as vehicle controls. The supernatants were collected for analysis using the Human Cytokine Antibody Array (ab133997, Abcam, Boston, MA) following the manufacturer’s instructions. Uncultured media was used as blank controls. This assay allows us to target 42 cytokines, including TPO, GM-CSF, G-CSF, IL-3, etc. (Fig. S10b). The chemiluminescence data were acquired using the ChemiDoc MP (BioRad) system, and the data were processed using ImageLab software (BioRad, version 6.0.1, Hercules, CA, USA). The data are indicated as fold changes and normalized with vehicle control after correcting for background intensity.

**Animal studies**

All *in vivo* studies were performed under approved animal care protocols and the standards of the Association for Assessment and Accreditation of Laboratory Animal Care.

To evaluate the anti-leukemia efficacy of co-targeting CXCR4/E-selectin and FLT3, we established a PDX AML mouse model by injecting AML patient tissue samples (3x10^6/mouse) into NSG mice that received irradiation treatment (250 mGy) one day prior to the cell injection. The PDX cells were from an AML patient who had experienced relapse after sorafenib+E6201+DAC treatment but was still sensitive to sorafenib ex vivo and had high levels of E-selectin-L and CXCR4 in vitro. The PDX cells were further expended in NSGS mice and collected from the spleen and BM when the mice reached 90% engraftment. These expended PDX cells also showed resistance to quizartinib *ex vivo*. The collected cells were injected into NSG mice (3.5x10^6 cells/mouse) via the tail vein. The mice received quizartinib (3 mg/kg) or GMI-1359 (40 mg/kg) from days 63 to 115. Leukemia cell engraftment was determined by measuring the hCD45-positive/mCD45-negative cell population in peripheral blood on the indicated days. Three mice in each group were humanely sacrificed on day 85 after PDX cell injection and the BM and spleen were collected for immunofluorescence staining, as described above, to assess leukemia cell infiltration in these organs. The remaining mice were closely monitored to determine their survival.

In another set of combination therapy of co-targeting CXCR4/E-selectin and FLT3, the PDX cells were injected into NSG mice (3x10^6/mouse). The mice received first-generation FLT3 inhibitor sorafenib (8 mg/kg) or GMI-1359 (40 mg/kg) when the leukemia cells reached 1%-2% engraftment in peripheral blood (from day 20 to 99). Leukemia cell engraftment in peripheral blood was determined by collecting peripheral blood samples from the mice and measuring hCD45-positive/mCD45-negative cells with flow cytometry on the indicated days. Three mice from each group were sacrificed (euthanasia with CO) and their BM samples were fixed in 10% neutral-buffered formalin solution at 4ºC overnight. Bone tissues were decalcified in 14% EDTA. The tissues were dehydrated, embedded in paraffin, and sectioned. After antigen retrieval, the slides were incubated with anti-mCD13 and –mCD41 antibodies overnight. Semi-quantitative analysis of mouse myelocytes and megakaryocytes by counting mCD13- and mCD41-positive cell numbers. Error bars are presented as the means ± standard deviation.

**Statistical analyses**

All data shown in the bar graph figures were generated from at least three independent experiments; error bars are presented as the means ± standard deviation. The statistical analyses were performed using an unpaired Student's *t*-test or one-way ANOVA (as indicated). A *p-value* ≤ 0.05 was considered statistically significant. The efficacy of the drug treatments on survival duration was estimated by the Kaplan-Meier method ^6^; log-rank statistics were used to test for differences in survival.

**References**

1. Zhang W, Gao C, Konopleva M, Chen Y, Jacamo RO, Borthakur G*, et al.* Reversal of Acquired Drug Resistance in FLT3-Mutated Acute Myeloid Leukemia Cells via Distinct Drug Combination Strategies. *ClinCancer Res* 2014 5/1/2014; **20**(9)**:** 2363-2374.

2. Zhang Q, Riley-Gillis B, Han L, Jia Y, Lodi A, Zhang H*, et al.* Activation of RAS/MAPK pathway confers MCL-1 mediated acquired resistance to BCL-2 inhibitor venetoclax in acute myeloid leukemia. *Signal Transduct Target Ther* 2022 Feb 21; **7**(1)**:** 51.

3. Tabe Y, Konopleva M, Munsell MF, Marini FC, Zompetta C, McQueen T*, et al.* PML-RARalpha is associated with leptin-receptor induction: the role of mesenchymal stem cell-derived adipocytes in APL cell survival. *Blood* 2004 3/1/2004; **103**(5)**:** 1815-1822.

4. Clodi K, Kliche K-O, Zhao S, Weidner D, Schenk T, Consoli U*, et al.* Cell-surface exposure of phosphatidylserine correlates with the stage of fludarabine-induced apoptosis in chronic lymphocytic leukemia (CLL) and expression of apoptosis-regulating genes. *Cytometry* 2000 2000; **40**(1)**:** 19-25.

5. Zhang W, McQueen T, Schober W, Rassidakis G, Andreeff M, Konopleva M. Leukotriene B4 receptor inhibitor LY293111 induces cell cycle arrest and apoptosis in human anaplastic large-cell lymphoma cells via JNK phosphorylation. *Leukemia* 2005; **19**(11)**:** 1977-1984.

6. Kaplan E, Meier P. Nonparametric estimation from incomplete observations. *J Am Stat Assoc* 1958 1958; **53:** 457-481.
