# Supplementary material for: FLT3 inhibitors upregulate CXCR4 and E-selectin ligands via ERK suppression in AML cells and CXCR4/E-selectin inhibition enhances anti-leukemia efficacy of FLT3-targeted therapy in AML
Source: Leukemia. 2023 Apr 21;37(6):1379–83. doi: 10.1038/s41375-023-01897-x (PMC10244167; doi:10.1038/s41375-023-01897-x)
Supplement: Supplementary file 3 — Supplementary figures [file 41375_2023_1897_MOESM3_ESM.pdf]

Supplementary Data:

Supplementary Table 1. Primary AML samples for checking CD44 and CXCR4 basal levels.

Case #	Diagnosis	BM Blasts	Cytogenetics	Mutations	Prior Rx	clinical response
Case 1	AML	84	46,XX[20]	FLT3-ITD, CEPBA, IDH1, NPM1, NRAS	Newly diadnosed	N/A
Case 2	AML	68	ND; Diploid OS PreRx	FLT3-ITD, DNMT3A, IDH1, NPM1	standard 3+7	CR
Case 3	AML	93	47,XY,+5[7] 46,XY[13]	FLT3-ITD, IDH1, NPM1 [CMS53]	Sorafenib, Crenolanib	Relapsed
Case 4	AML	79	46,XX[20]	FLT3-ITD, DNMT3A, NPM1 [CMS53]	ICE,HD AC,Clofa+Ara-	R/R
Case 5	AML	96	46,XY,del(3)(q13.1),add(6)(p25),del(9)(p21p24),del(11)(p11.2)[17]; Random chgs[3]	FLT3-ITD, DNMT3A, NPM1, TP53	IA+IL11(CR:5M),Triapine+AC, Cloretazine,BID FA,Gemcit+Mitox	R/R
Case 6	AML	84	46,XX,t(3;21)(p25;q22)[11];46,idem,der(19)t(8;19)(q13;p13.3)[3]; 47,idem,+8[3];46,XX,der(3)t(3;21)(p25;q22),der(21)t(3;21)t(8;21)(q11.2;p11.2)[6]; 46,XX,der(9)t(8;9)(q13;q34)[7]	FLT3-ITD [CMS28]	3+7,Mitox+HD AC,Clofa+Etop+Ctx(CR:4M,SCT),Aza+DLI, sorafenib	Refractory

N/A, not available; CR, complete response; R/R, relapsed/refractory.

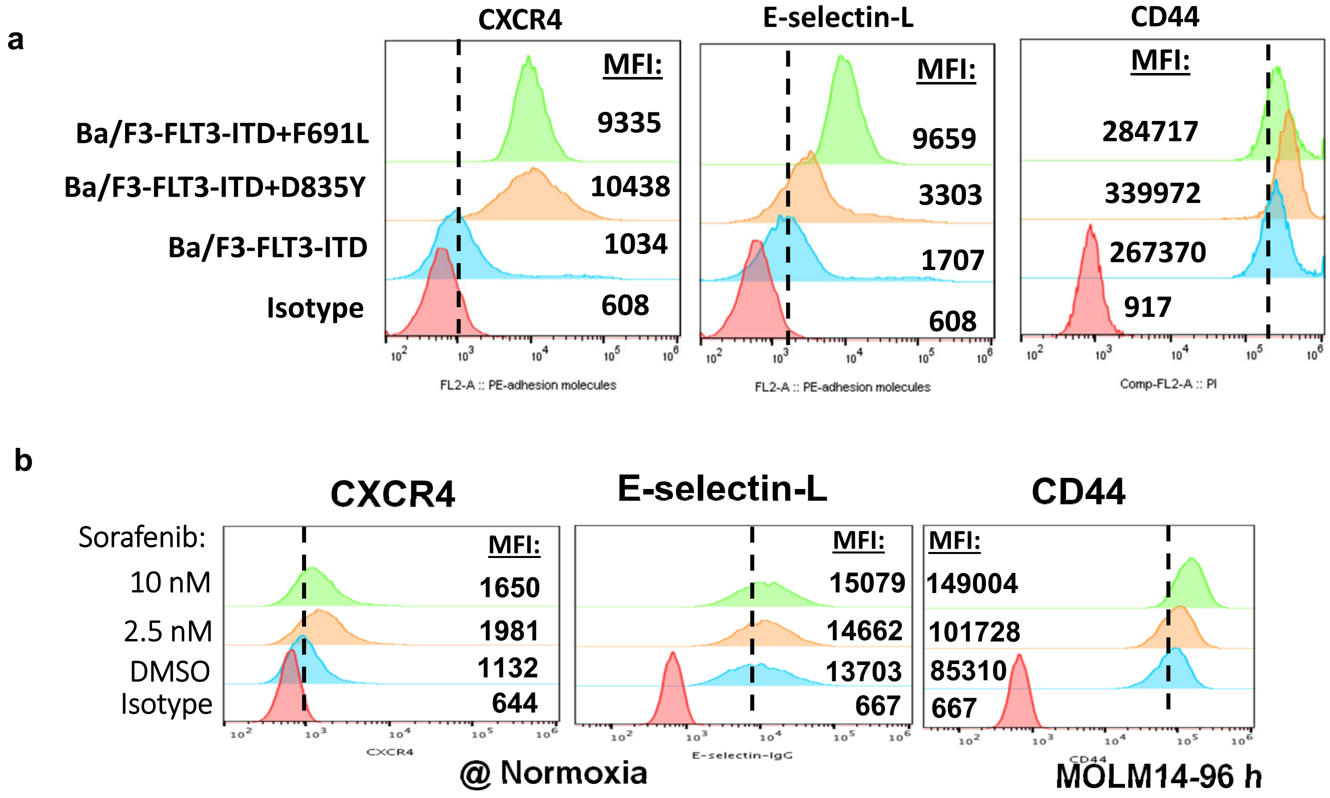

Fig. S1. (a) FLT3i-resistant cell lines Ba/F3-*FLT3*-ITD+D835Y, Ba/F3-*FLT3*-ITD+F691L, and FLT3i-sensitive cell line Ba/F3-*FLT3*-ITD were used for investigating their basal levels of CXCR4, E-selectin-L, and CD44 on the cell surface by staining with anti-CXCR4-PE, E-selectin-IgG-PE, and anti-CD44-PE antibodies, and then measured median fluorescence intensity (MFI) using flow cytometry. (b) MOLM14 cells were exposed to FLT3i sorafenib for 96 h and the levels of CXCR4, E-selectin-L, and CD44 were determined using flow cytometry.

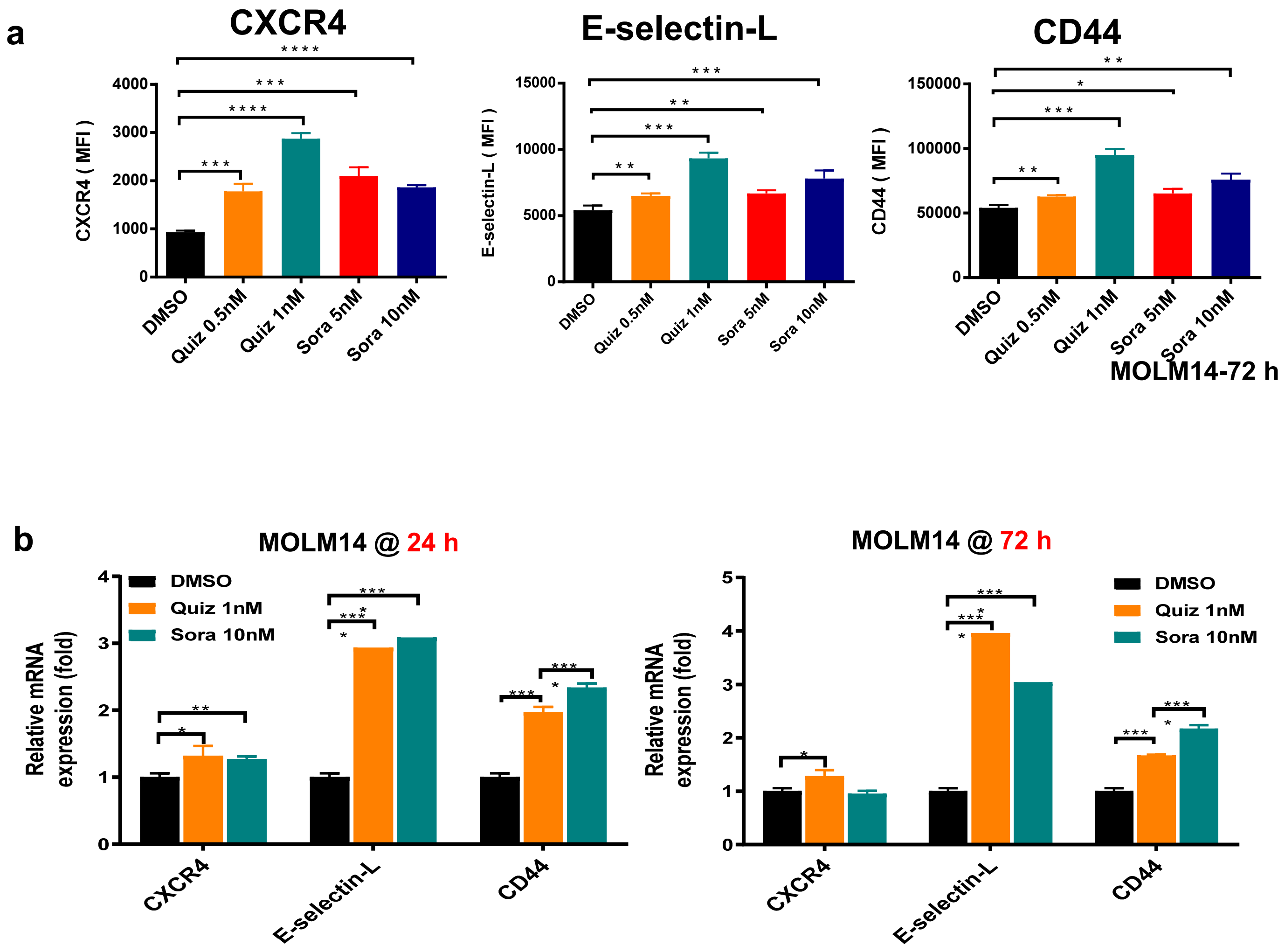

Fig. S2. MOLM14 cells were exposed in indicated concentrations of sorafenib or quizartinib for 24 or 72 h. The surface protein levels of CXCR4, E-selectin-L and CD44 were measured by flow cytometry (a) and mRNA levels were measured using qPCR (b). The numbers of x-axes indicated relative mean fluorescent intensity (MFI) of the proteins or mRNA levels (fold) of normalizing with housekeeping gene GAPDH at DMSO groups Error bars presented as the means ± standard deviation from three independent experiments.

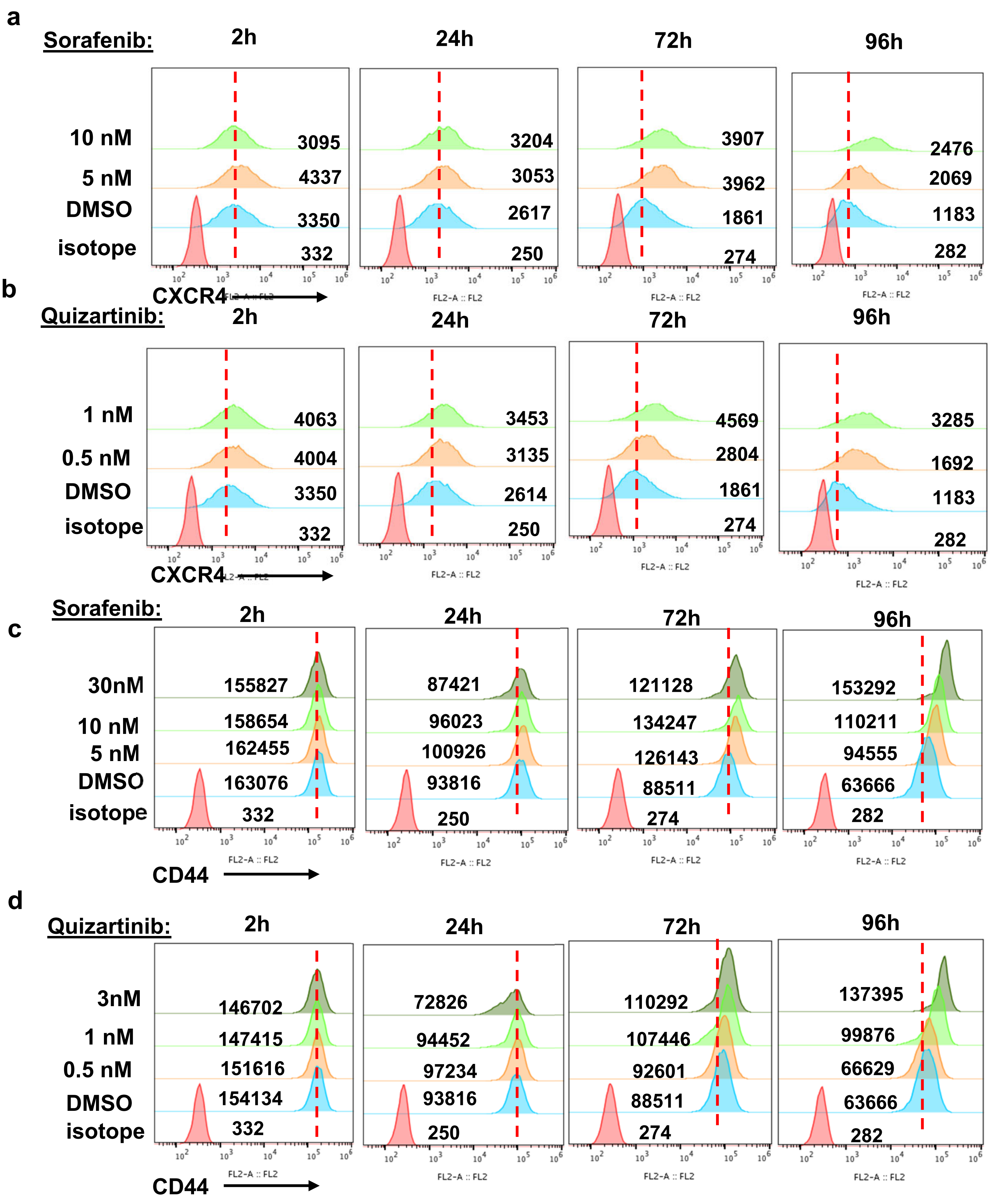

Fig. S3. MOLM14 cells were exposed in indicated concentrations of FLT3i sorafenib or quizartinib for 2, 24, 72 and 96 h. Cell surface CXCR4 (a, b) and CD44 (c, d) were measured using flow cytometry after staining with their corresponding antibodies Isotype-PE was as background control.

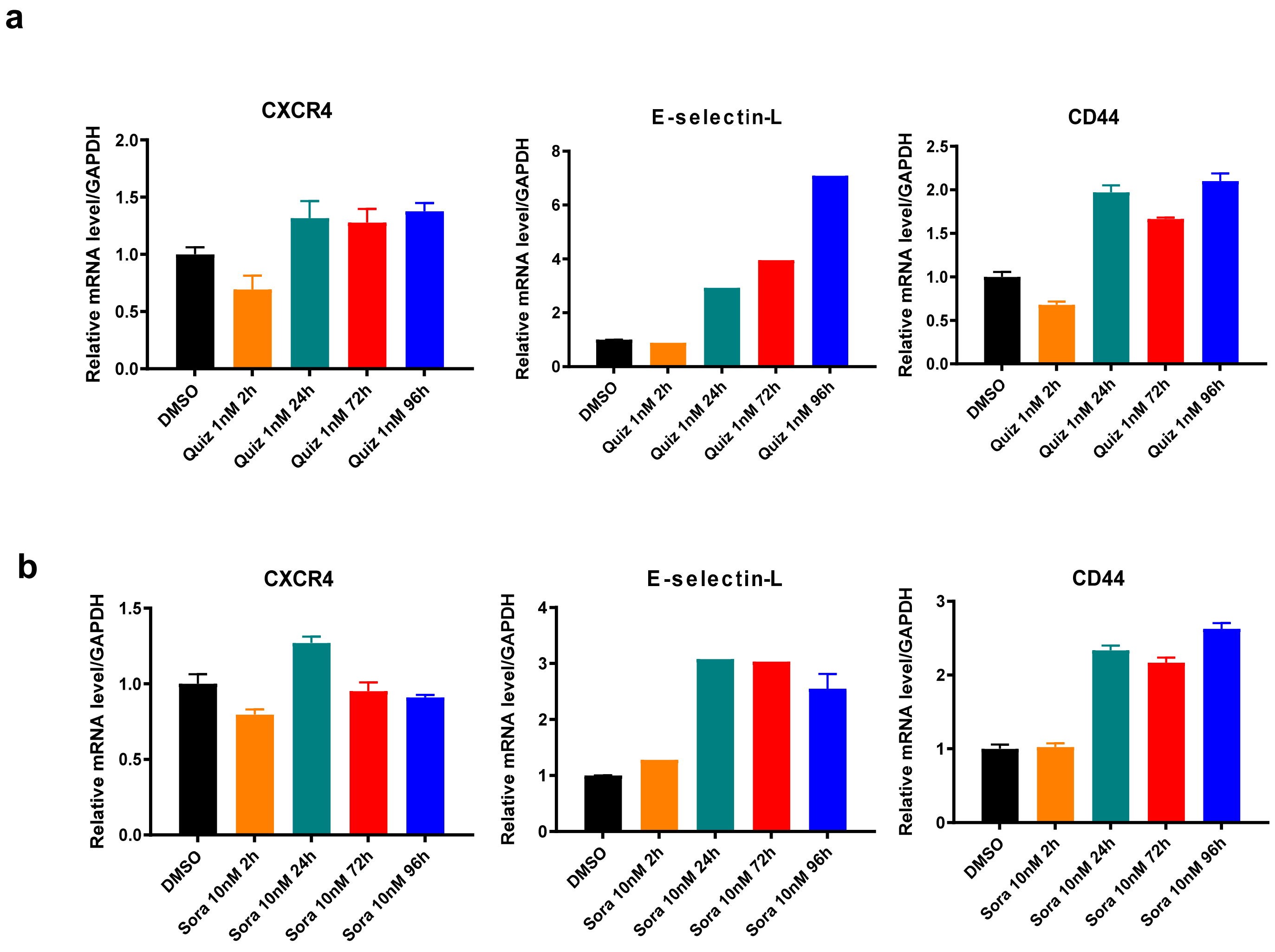

Fig. S4. MOLM14 cells were exposed in indicated concentrations of quizartinib (a) or sorafenib (b) for 2, 24, 72 and 96 h. The mRNA levels of CXCR4, E-selectin-L and CD44 were measured using qPCR. The numbers of *x*-axes indicated mRNA levels (fold) of normalizing with housekeeping gene GAPDH at DMSO groups Error bars presented as the means ± standard deviation from three independent experiments.

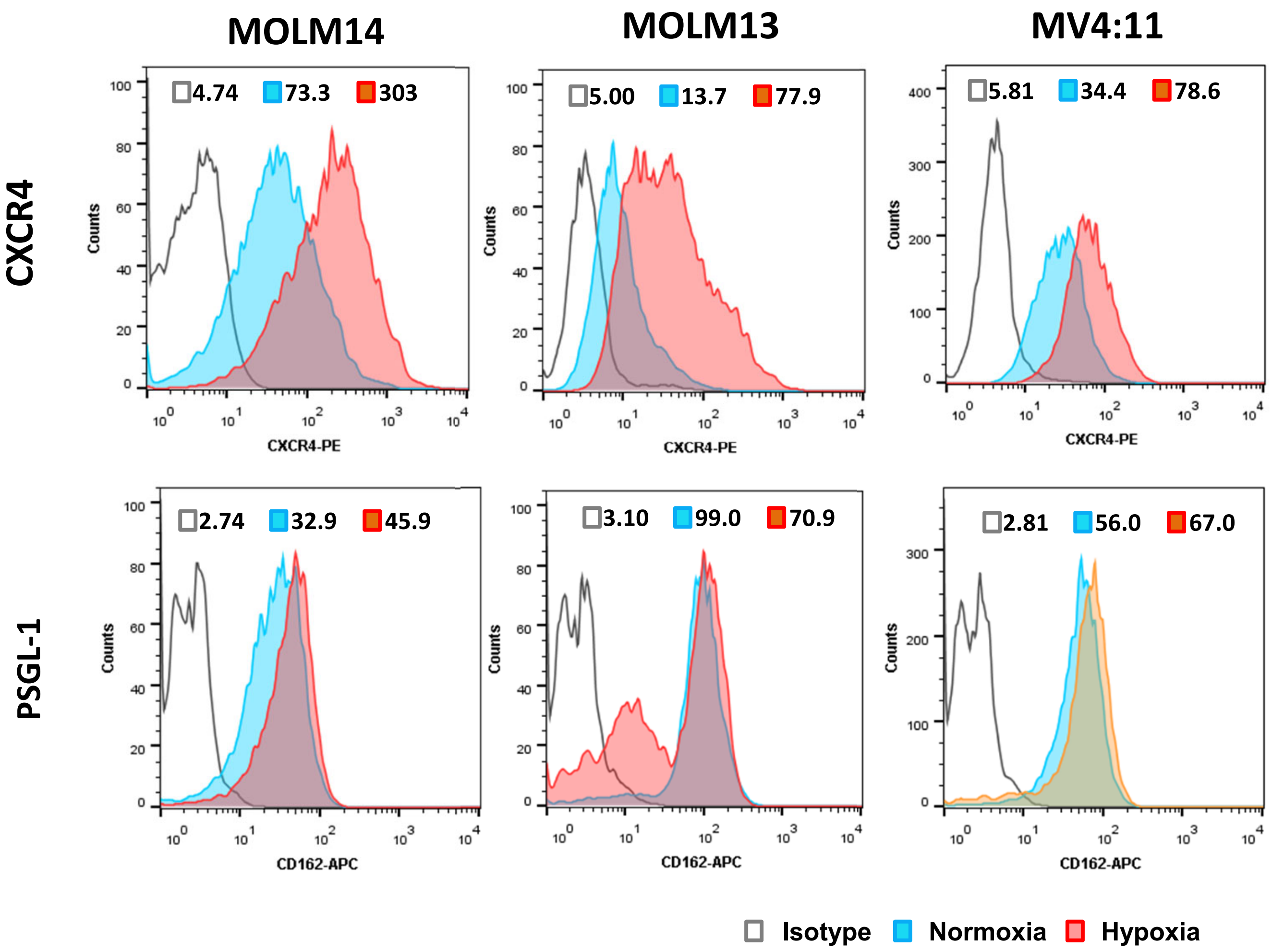

Fig. S5. FLT3 ITD mutated human leukemia cell lines MOLM13, MOLM14 and MV:11 were cultured in either normoxia or hyposia conditions for 48 h. Cell surface CXCR4 (a), and E-selectin-L (PSGL-1) (b) were measured using flow cytometry after staining with their corresponding antibodies Isotype-PE was as background control. The numbers indicated mean fluorescence intensity (MFI).

MAK/ERK inhibition:

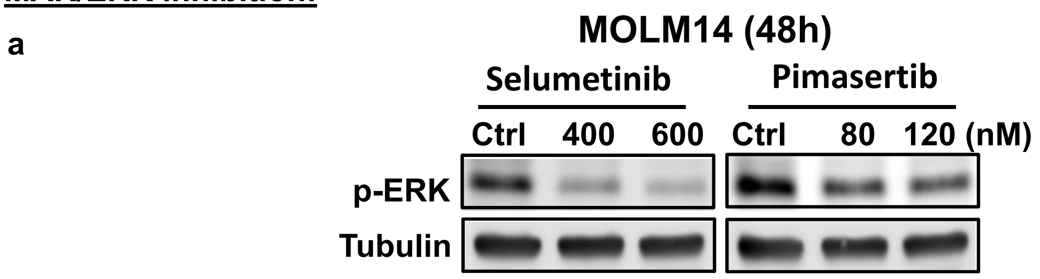

mTOR inhibition:

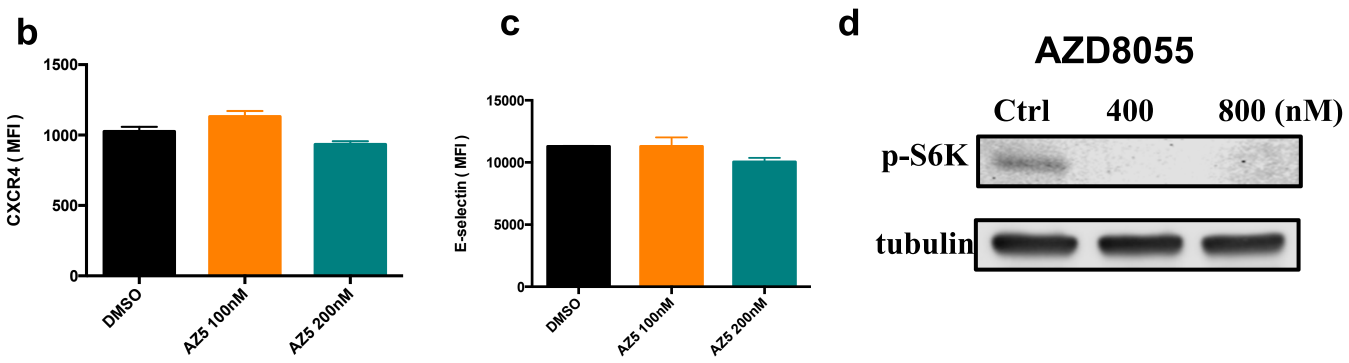

Stat5 inhibition:

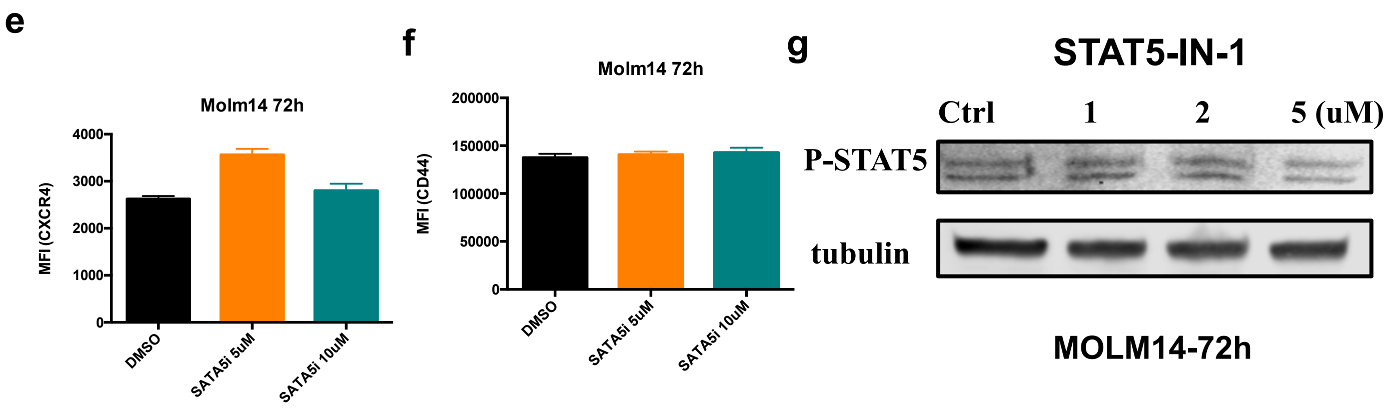

Fig. S6. MOLM14 cells were exposed to indicated concentrations of (a) MAK/ERK inhibitors selumetinib and pimasertib, (b-d) mTORi AZD8055 or (e-g) STAT5i STAT5-IN-1 for 72 h. the correlated phosphorylated proeteins were measured with immunoblotting, and surface CXCR4 and E-selectin-L (CD44) were measured by using flow cytometry.

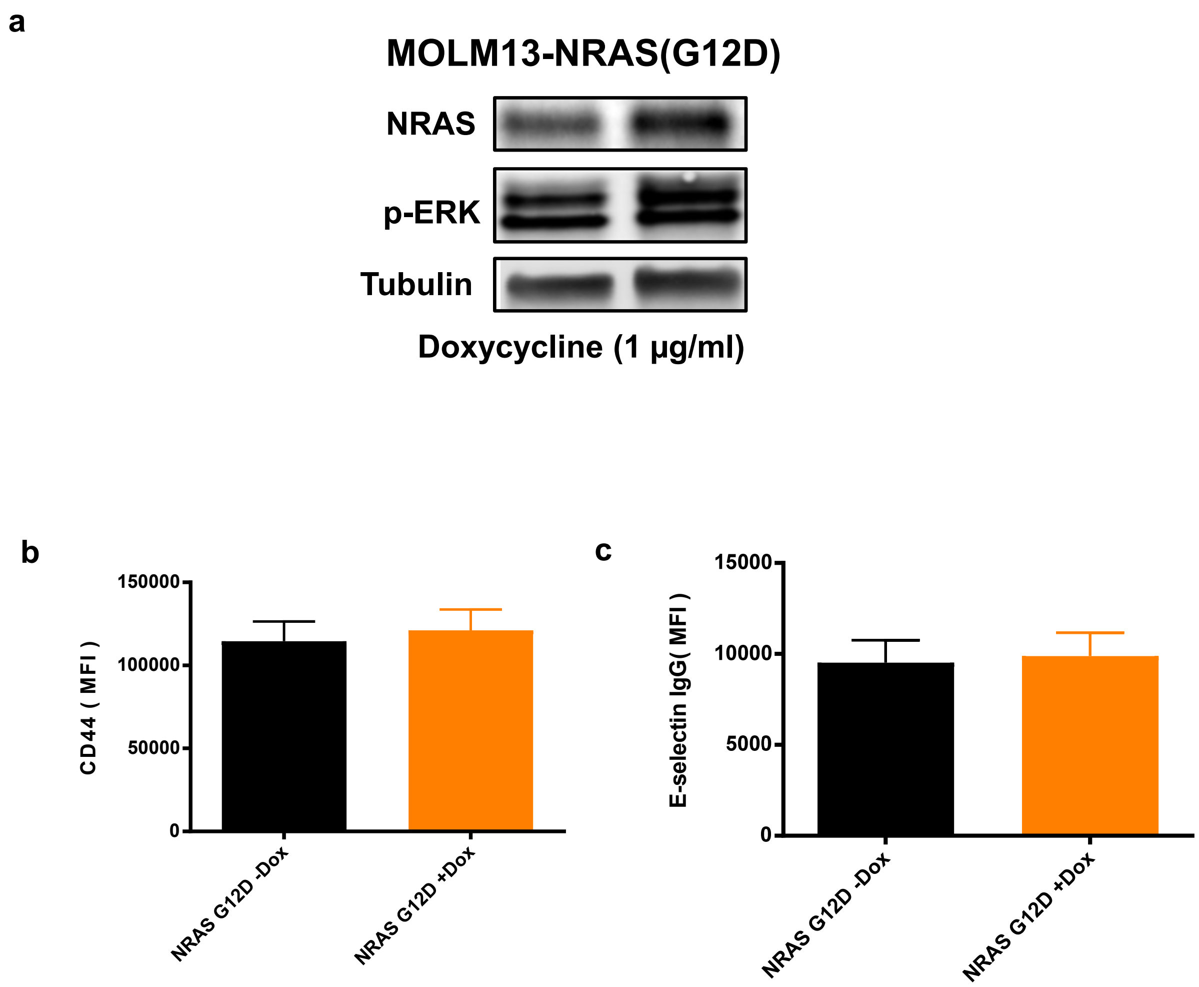

Fig. S7. Dox-inducible MOLM13-NRAS(G12D) mutant cells were cultured in the presence/absence Dox for 72 h. NRAS and phospho-ERK levels were determined using immunoblotting (a) and the MFI of the cell surface (b) CD44 or (c) E-selectin-L were measured using flow cytometry..

a

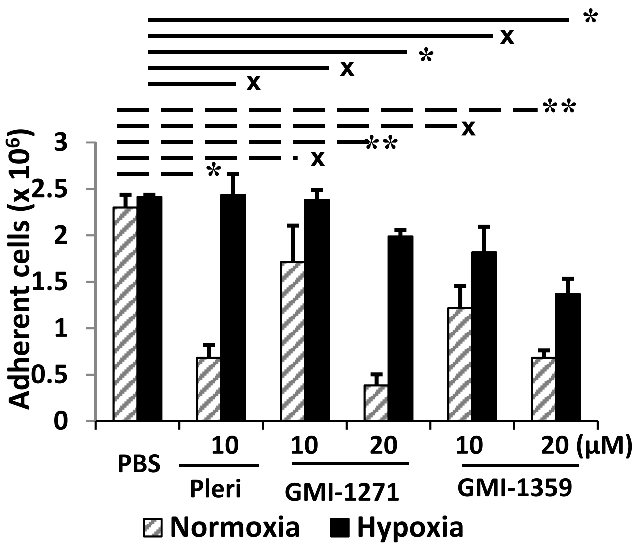

b

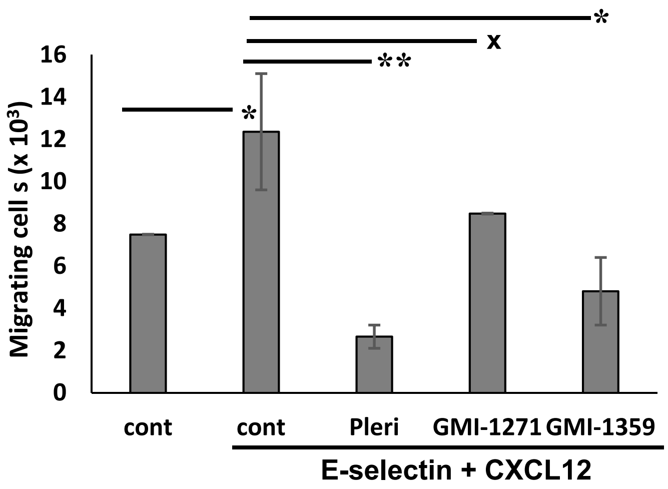

Fig. S8. (a) The MSC and EC feeder layer was pretreated with TNFα (100 ng/mL) for 24 h; MOLM14-GFP cells were pretreated with the indicated drugs for 2 h and then seeded on the feeder layer for an additional 20-h co-culture in normoxia or hypoxia conditions. PBS treatment was used as a control. The adherent leukemia cells were trypsinized and cell numbers were determined using flow cytometry by calculating the GFP+ population with counting beads. (b) MV4:11-GFP cells were pretreated with the indicated drugs for 1 h and were added into inner chambers and either co-cultured with seeded into E-selectin/CXCL12-coated wells for an additional 3-h culture. Migrated GFP+ cells in outer chambers were collected by trypsinization and counted using flow cytometry with counting beads. Pleri: plerixafor.

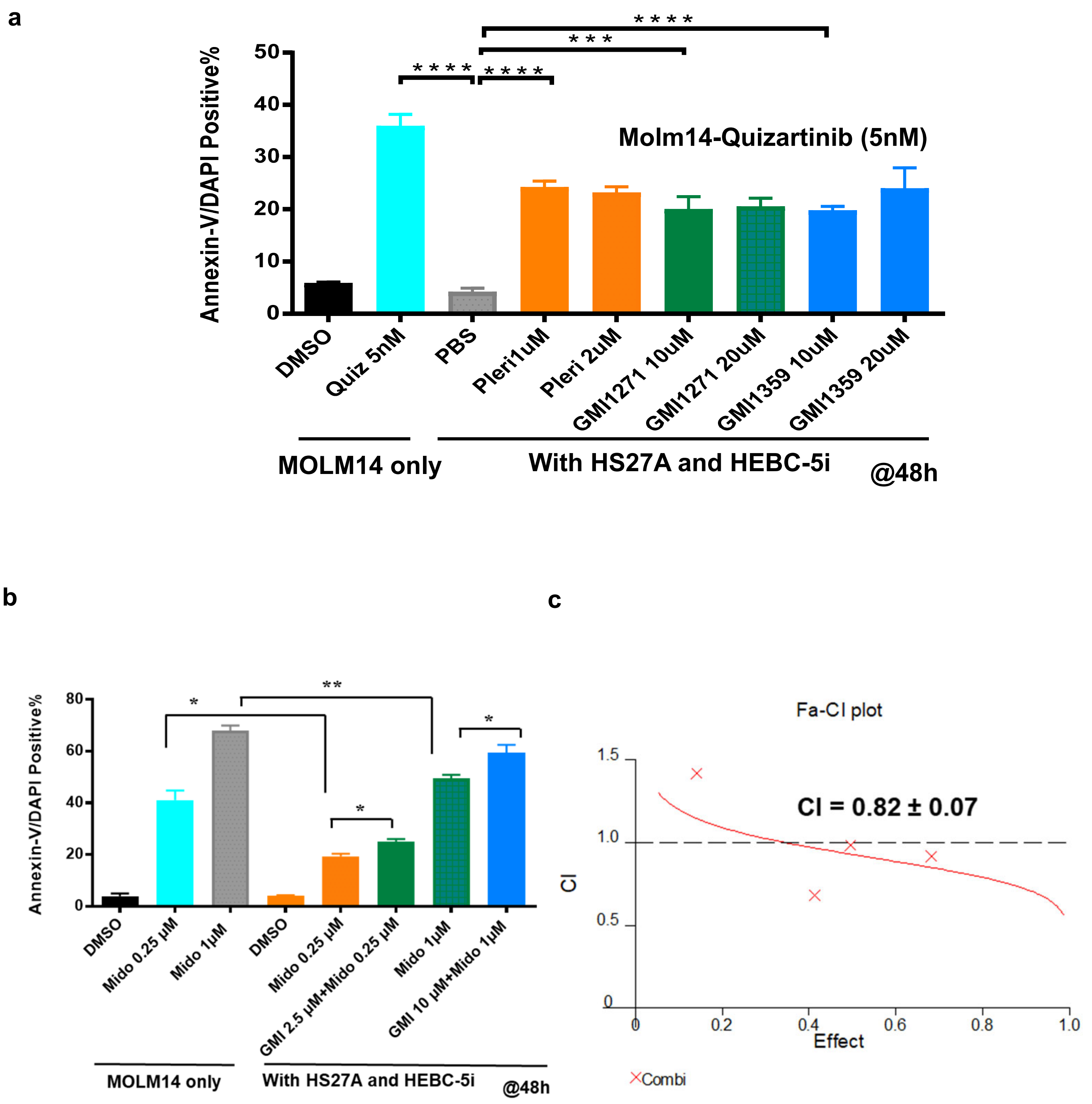

Fig. S9. MOLM14 cells were pretreated with plerixafor or GMI-1271 or GMI-1359 for 2 h and treated with FLT3i quizartinib (a) or FDA-approved midostaurin (b) for additional 48 in co-culture with human the MSC/EC (HS27A/HEBC-5i) feeder layer. Cell apoptosis induction was determined using flow cytometry by counting the Annexin V+/CD90- DAPI+ population. (c) Combination index (CI) of GMI-1359 with midostaurin treatment was calculated using CalcuSyn software (BioSoft, Cambridge, UK). Pleri: plerixafor; Mido: midostaurin; GMI: GMI-1359.

Fig. 6

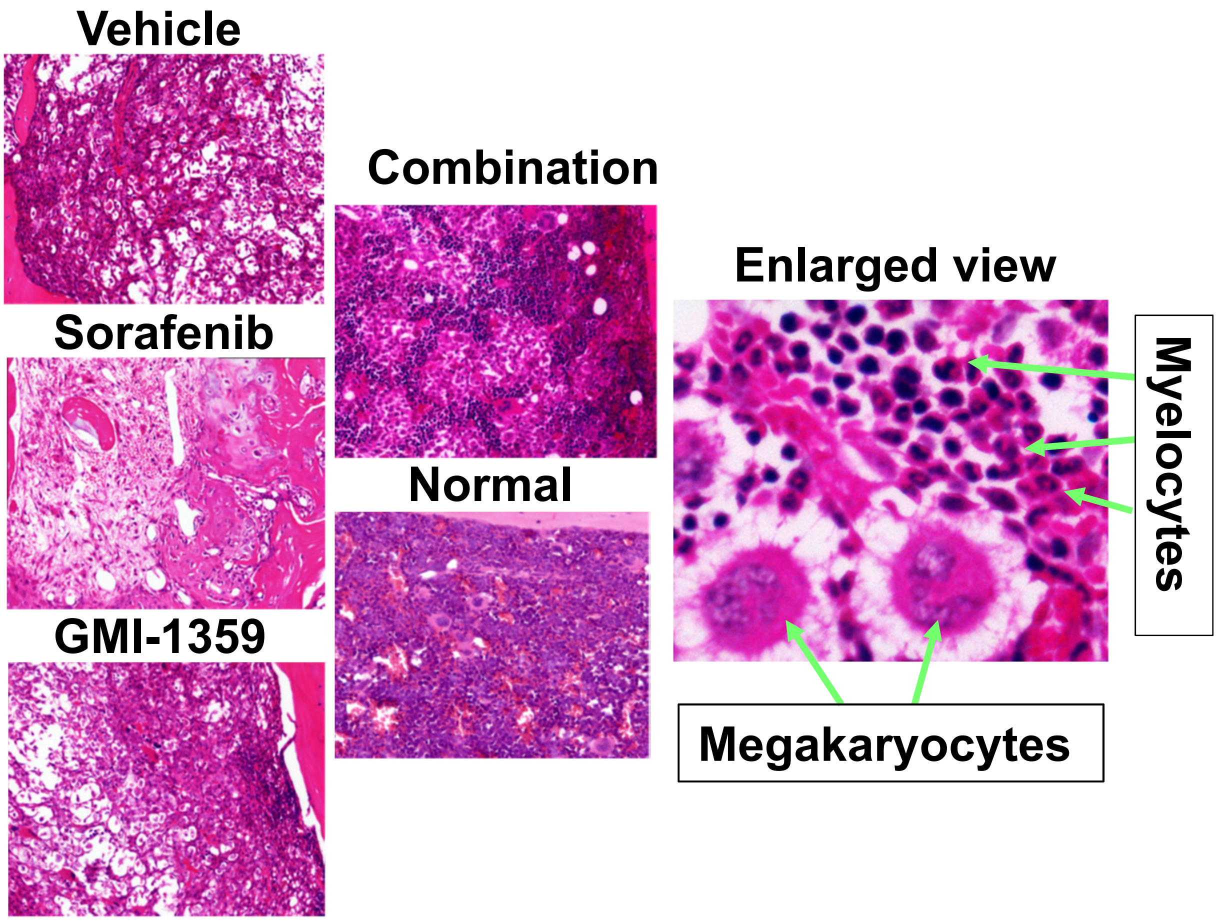

Fig. S10. BM samples from a PDX murine model, which received sorafenib and/or GMI-1359 administration for 53 days, were stained with H&E. Morphological analysis of megakaryocytes and myelocytes were performed under microscopy. Enlarged view showed the morphological features of the megakaryocytes and myelocytes (green arrows).

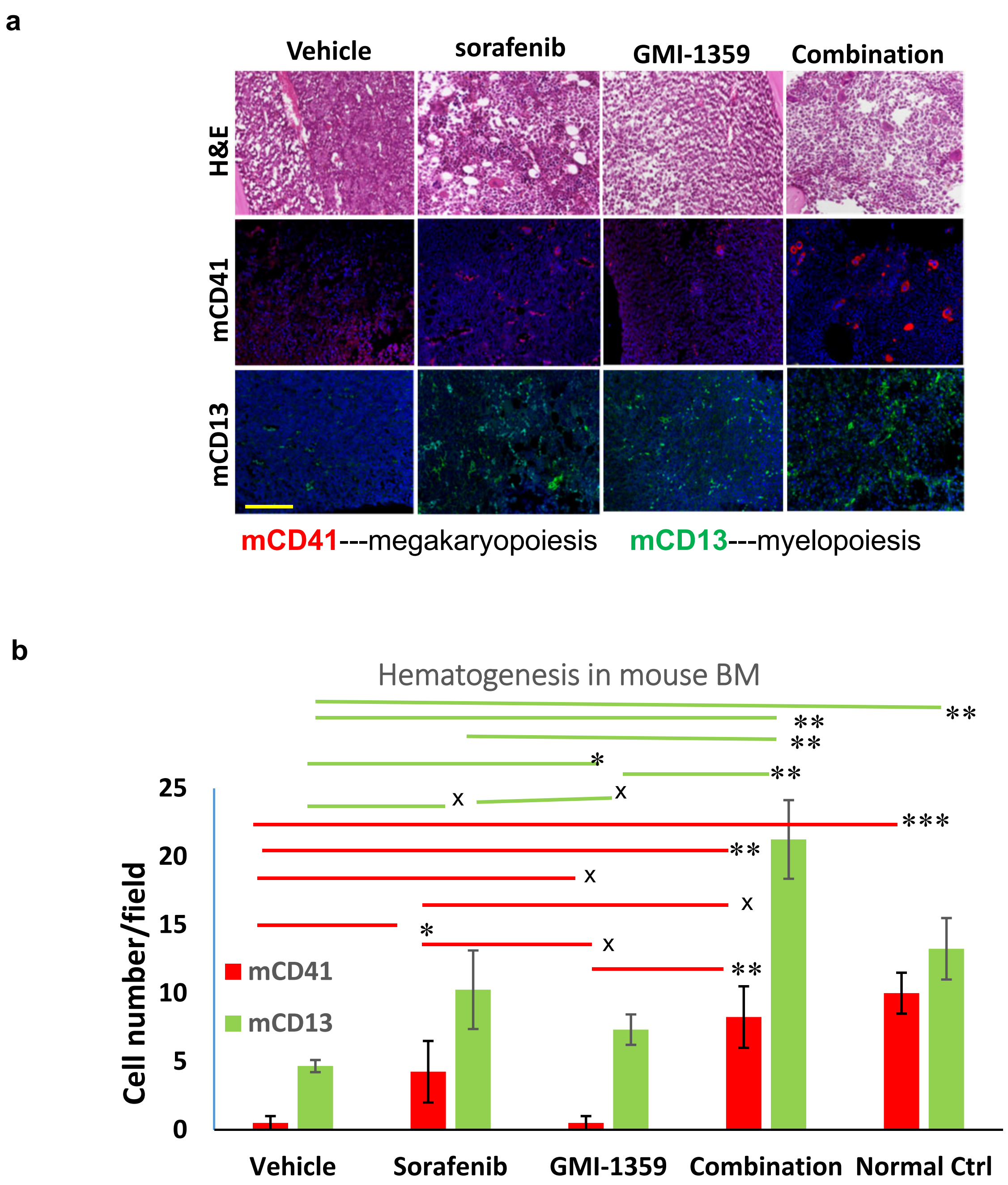

Fig. S11. (a) NSG mice engrafted with PDX AML cells received sorafenib and/or GMI-1359 (53 days of treatment). Histological sections of BM samples were stained with H&E and anti-mouse CD41 (red) and anti-mouse CD13 (green) immunofluorescence. Bar = 100 μm. (b) semiquantitative analysis of immunofluorescence images from BM samples by counting mCD41- and mCD13-positive cell numbers. Error bars are presented as the means ± standard deviation. The asterisks indicate the level of statistical significance. * = $p < 0.05$; ** = $p < 0.01$; *** = $p < 0.001$; **** = $p < 0.0001$; and x = not statistically significant.

a

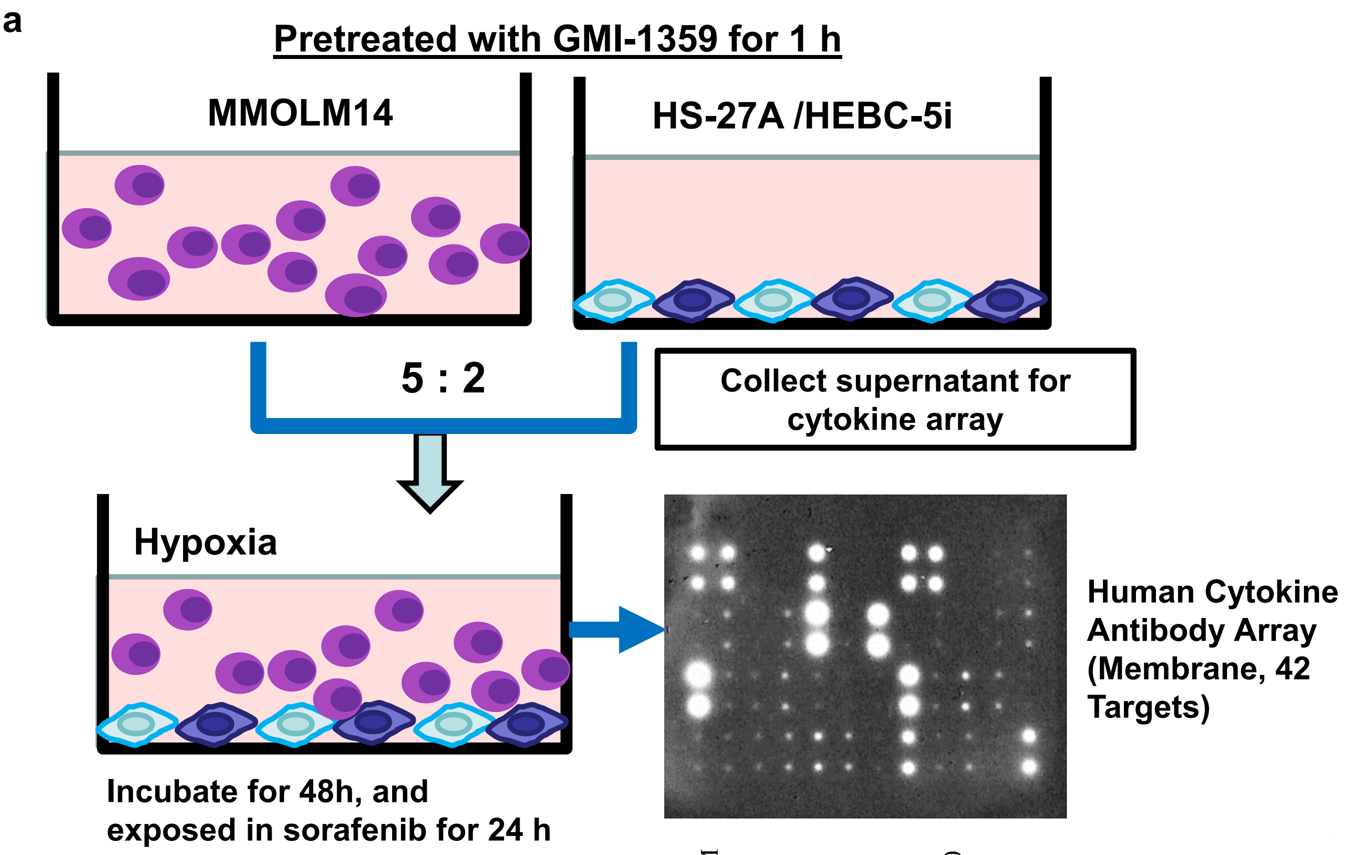

b

	IL-6	RANTES	IL-8	MCP-1	ENA-78	GRO	GRO-A	VEGF	Angiogenin	IL-1B	IL-2	IL-1A	IFN-r	SDF-1
Vehicle	156.99	139.13	138.98	118.60	72.52	63.43	62.93	50.54	36.96	30.45	28.92	24.82	23.95	23.57
GMI-1359	115.26	90.94	89.21	88.37	40.79	35.46	34.86	29.09	25.12	18.66	36.08	12.90	17.89	14.32
Sorafenib	95.91	90.13	113.70	114.13	46.43	52.29	46.93	42.56	17.89	4.60	64.30	3.52	8.60	15.82
Comb	137.05	122.80	146.82	150.54	66.84	56.55	48.26	42.91	25.76	7.12	48.39	6.40	4.34	15.46

	TARC	TNF-B	EGF	IL-3	IL-15	TNF-A	IGF-I	Oncostatin M	IL-5	I-309	Leptin	IL-7	MCSF	SCF
Vehicle	20.04	19.84	19.36	19.20	19.12	18.89	18.52	16.93	15.58	13.18	13.14	12.92	12.72	10.66
GMI-1359	14.42	6.66	9.17	15.45	10.07	7.93	13.97	6.28	8.08	6.23	9.17	8.62	10.56	6.01
Sorafenib	6.78	4.29	3.97	35.21	6.11	9.09	5.20	9.28	9.01	5.32	4.76	10.75	7.69	13.41
Comb	4.55	3.64	5.64	34.86	3.01	1.74	8.52	8.25	20.61	5.50	2.86	14.72	12.91	8.74

	IL-10	IL-12 p40/p70	IL-13	MCP-2	MCP-3	TGF-B1	MDC	PDGF BB	IL-4	GCSF	MIG	MIP-1D	GMCSF	ThPO
Vehicle	10.15	10.06	9.67	9.07	8.27	7.75	5.92	5.39	4.49	2.79	1.95	1.86	1.65	0.57
GMI-1359	5.09	3.52	5.36	8.05	7.21	14.26	9.87	4.91	4.80	0.87	4.79	2.28	1.63	1.64
Sorafenib	11.94	10.63	8.02	15.90	5.42	2.17	5.94	8.40	11.04	5.93	5.92	10.23	5.98	6.58
Comb	13.31	9.60	6.28	15.60	10.75	0.53	2.54	0.97	17.58	5.92	7.77	10.77	5.52	2.62

High — Low

Fig. S12. (a) Schematic diagram of Cytokine antibody array and membrane result after development of antibody immunoblotting. (b) MOLM14 cells were exposed in sorafenib and/or GMI-1359 for 24 h in hypoxia condition. The levels of 42 targeted cytokines were determined by calculating dots intensity. The data were indicated as fold changes and normalized with vehicle control.

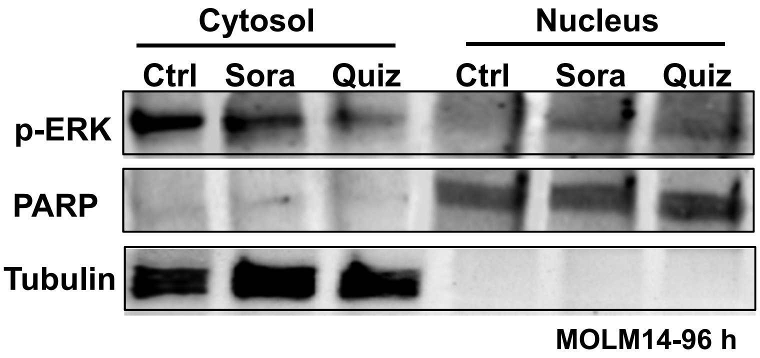

Fig. S13. MOLM14 cells were exposed to sorafenib (10 nM) or quizartinib (1 nM) for 96 h. The protein levels in cytosol and nuclear fractions were determined by using immunoblotting.
